# Diverse Clinical Isolates of *Mycobacterium tuberculosis* Develop Macrophage-Induced Rifampin Tolerance

**DOI:** 10.1093/infdis/jiy710

**Published:** 2019-02-07

**Authors:** Kristin N Adams, Amit Kumar Verma, Radha Gopalaswamy, Harresh Adikesavalu, Dinesh Kumar Singhal, Srikanth Tripathy, Uma Devi Ranganathan, David R Sherman, Kevin B Urdahl, Lalita Ramakrishnan, Rafael E Hernandez

**Affiliations:** 1 Center for Global Infectious Diseases Research, Seattle Children’s Research Institute, Center for Infectious Diseases Research, Seattle, Washington; 2 Department of Pediatrics, University of Washington, Seattle, Washington; 3 Molecular Immunity Unit, Department of Medicine, University of Cambridge, United Kingdom; 4 National Institute for Research in Tuberculosis, Chennai, India

**Keywords:** Tuberculosis, drug efflux, Beijing lineage, *Rv1258c*, antibiotic tolerance

## Abstract

The *Mycobacterium tuberculosis* lineage 4 strains CDC1551 and H37Rv develop tolerance to multiple antibiotics upon macrophage residence. To determine whether macrophage-induced tolerance is a general feature of clinical *M. tuberculosis* isolates, we assessed macrophage-induced drug tolerance in strains from lineages 1–3, representing the other predominant *M. tuberculosis* strains responsible for tuberculosis globally. All 3 lineages developed isoniazid tolerance. While lineage 1, 3, and 4 strains developed rifampin tolerance, lineage 2 Beijing strains did not. Their failure to develop tolerance may be explained by their harboring of a loss-of-function mutation in the Rv1258c efflux pump that is linked to macrophage-induced rifampicin tolerance.

## Teaser

*Mycobacterium tuberculosis* develops tolerance to multiple antibiotics when residing in host macrophages. We demonstrate that macrophage-induced tolerance to rifampin is common across major lineages of *M. tuberculosis*, except for Beijing-family lineage 2 strains.

*Mycobacterium tuberculosis* enters host macrophages shortly after infection and resides within granulomas, organized macrophage aggregates, for much of its life cycle [1]. Previously, we and others showed that *M. tuberculosis* develops tolerance to multiple first-line and second-line antituberculosis drugs soon after infecting macrophages [2–4]. Moreover, we found that macrophage-induced tolerance to rifampin is mediated via *Tap* (*Rv1258c*), a major facilitator superfamily efflux pump [2]. *Rv1258c* expression is induced when bacteria reside within cultured human macrophages [5], as well as in bacteria in sputum from patients with tuberculosis who are undergoing treatment with a rifampin-containing regimen [6]. These observations suggest that macrophage-induced tolerance to rifampin mediated by *Rv1258c* may contribute to drug tolerance observed in patients.

Based on genomic differences, *M. tuberculosis* is broadly categorized into multiple lineages associated with distinct phenotypes with regard to mutability, drug susceptibility, immunogenicity, and virulence [7]. The vast majority of tuberculosis worldwide (>90%) is caused by *M. tuberculosis* lineages 1 (Indo-Oceanic), 2 (East Asian), 3 (East African–Indian), and 4 (Euro-American; Supplementary Figure 1) [7]. Our prior observations of macrophage-induced tolerance were made in H37Rv and CDC1551, both of which represent lineage 4 strains. While lineage 4 is perhaps the most widely distributed geographically, it accounts for only approximately 11% of the global tuberculosis burden (Supplementary Figure 1*B*) [7]. Therefore, we sought to determine whether macrophage-induced tolerance to isoniazid and rifampin is a shared feature across the other 3 lineages that are predominant in areas of high tuberculosis burdens [7]. In addition, because the Beijing-subgroup lineage 2 strains harbor an inactivating frameshift mutation in *Rv1258c* [8], we were interested to see whether they develop rifampin tolerance. Furthermore, Rv1258c also facilitates bacterial growth within macrophages [2, 9], so we assessed both Beijing and non-Beijing strains for growth within macrophages.

## METHODS

### Bacterial Strains

The sources and antibiotic susceptibilities of the strains used are detailed in Supplementary Table 1. Bacteria were grown to mid-log phase in Middlebrook 7H9 medium (Becton Dickinson) with 0.05% Tween-80 and albumin, dextrose, and catalase (Middlebrook ADC Enrichment, Becton Dickson) before infection.

### Macrophage Growth and Infection

THP-1 cells (ATCC) were grown in Roswell Park Memorial Institute 1640 medium, supplemented with 10% fetal bovine serum and 2 mM l-glutamine (Sigma) in a 37°C incubator with 5% CO_2_. A total of 5 × 10^5^ THP-1 cells were differentiated into wells of 24-well plates with 100 nM phorbol 12-myristate 13-acetate (PMA; Sigma) for 48 hours, and then medium was replaced with fresh medium without PMA 24 hours before infection. The differentiated cells were infected at a multiplicity of infection of 1 for 2 hours. Cells were washed with medium, and 6 μg/mL streptomycin (Sigma) was added to medium for the remainder of the intracellular growth phase, to eliminate extracellular bacteria; this was defined as the start of infection. Medium was changed every 48 hours. For intracellular growth inhibition assays, verapamil HCl (40 μg/mL; Sigma) was added to the medium 48 hours after infection, and streptomycin was omitted.

### Macrophage-Induced Tolerance Assay

The work flow for this assay is depicted in Supplementary Figure 2. Briefly, THP1 cells were infected as described above and then lysed 2 or 96 hours after infection to release the bacteria, using the following protocol. First, cells were washed briefly once with phosphate-buffered saline and then with water. Cells were then incubated with 100 μL of water per well at 37°C for 15 minutes. Then, 900 μL of 7H9 medium (supplemented with Middlebrook ADC and 0.05% Tween-80) was added, and the well bottoms were scraped with a pipette tip to ensure complete macrophage lysis, which was confirmed by microscopy. Serial dilutions of 150 μL of cell lysates were made in phosphate-buffered saline and plated on 7H10 agar (Becton Dickson) to obtain the initial colony-forming units (CFU). To measure antibiotic killing, 500 μL of cell lysate was treated with the indicated antibiotic (ie, rifampin 1 μg/mL or isoniazid 0.6 μg/mL; Sigma) for 48 hours at 37°C, before undergoing serial dilution and plating on 7H10 agar. Percentage survival was determined by dividing the number of CFU after antibiotic treatment by the number before treatment.

### Intracellular Growth Assay

Infected cells were washed twice with PBS and incubated with 100 μL of 0.1% Triton X-100 for 10 minutes. Then, 900 μL of phosphate-buffered saline was added, and the wells were scraped with a pipette tip. Dilutions of cell lysates were plated on 7H10 agar as described above.

### Statistical Analyses

GraphPad Prism, version 6.0, was used for statistical analyses. Means were compared via the *t* test.

## RESULTS

### Macrophage-Induced Antibiotic Tolerance Occurs Across Predominant *M. tuberculosis* Lineages

Working at 2 sites, Seattle Children’s Research Institute (Seattle, WA) and the National Institute for Research in Tuberculosis (Chennai, India), we used a panel of *M. tuberculosis* strains representing lineages 1–4 assembled from previously published strains (at the Seattle site) and from recent clinical isolates (at the Chennai site; Supplementary Table 1). All strains were confirmed to be susceptible to both isoniazid and rifampin, except for strain NIRT203, which was resistant to isoniazid (Supplementary Table 1). The lineage 2 Beijing strains were confirmed to harbor the previously described frameshift mutation in *Rv1258c*, and this mutation was absent in all other strains, including the lineage 2 non-Beijing isolate M4100A (Supplementary Table 1).

**Figure 1.**
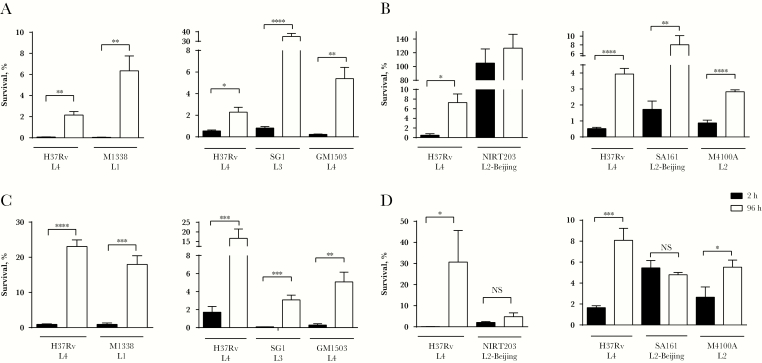
Macrophage-induced tolerance to rifampin is common across clinical lineages of *Mycobacterium tuberculosis.* THP-1 macrophages were infected with H37Rv (reference strain) or clinical strains as indicated and lysed 2 hours (black bars) or 96 hours (white bars) after infection. The released bacteria were treated for an additional 48 hours with 0.6 μg/mL isoniazid (*A* and *B*) or 1 μg/mL rifampicin (*C* and *D*) before enumeration of colony-forming units (CFU). Results are from 1 representative of 3 (*A*, *C*, and *D*) or 2 (*B*) independent experiments, which are defined as experiments set up on different days with different cultures of bacteria and THP-1 cells. Error bars represent standard deviations. Significance testing was performed using the *t* test. *P < .05, **P < .01, ***P < .001, and **** *P* < .0001, by the *t* test.

To assess development of macrophage-induced antibiotic tolerance, THP-1 macrophages were infected with the clinical *M. tuberculosis* strains and then lysed 2 and 96 hours after infection. Macrophage lysates were treated with antibiotics for 48 hours, and tolerance to antibiotics was assessed by comparing the number of CFU at lysis to the number of surviving CFU after antibiotic treatment (Supplemental Figure 2*A*). Macrophage-induced tolerance was defined as a statistically significant increase (*P* ≤ .05) in the fraction of bacteria surviving antibiotic treatment between the 2-hour and 96-hour time points. All of the isoniazid-susceptible strains developed macrophage-induced tolerance to isoniazid (Figure 1A and 1B), while NIRT203 was resistant to killing by isoniazid, as expected. Strains from lineages 1, 3, and 4 developed tolerance to rifampin (Figure 1C). The capacity of lineage 2 *M. tuberculosis* strains to develop tolerance to rifampin was variable. Both lineage 2 Beijing strains failed to develop rifampin tolerance (Figure 1D). In contrast, M4100A, a non-Beijing lineage 2 strain, developed macrophage-induced rifampin tolerance (Figure 1D).

### Lineage 2 Beijing Strains Grow Normally in Macrophages

In the CDC1551 strain, Rv1258c mutants not only fail to develop macrophage-induced rifampin tolerance, but also are defective for early growth in macrophages [2, 9]. However, Beijing strains do not exhibit a macrophage growth defect; indeed, many of them grow more rapidly in macrophages than non-Beijing isolates [10]. This may be one of the reasons that some Beijing strains have been found to be hypervirulent in animal infection models and are spreading globally [7, 11].

**Figure 2.**
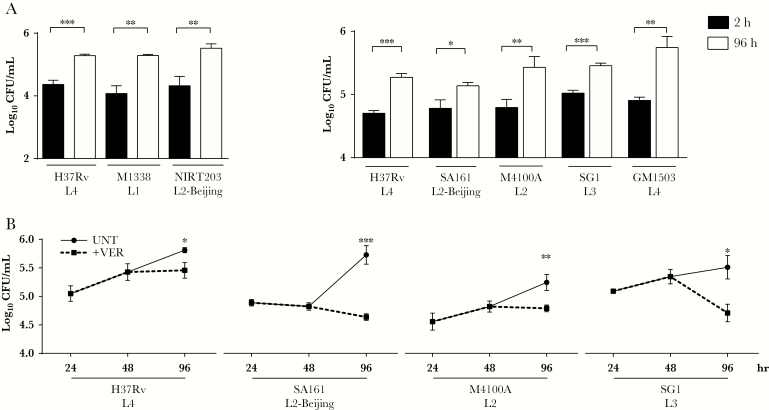
Beijing lineage strains of *Mycobacterium tuberculosis* are not compromised for early macrophage growth and are susceptible to intracellular verapamil treatment. *A*, THP-1 macrophages were infected with H37Rv or clinical strains of *M. tuberculosis* as indicated and lysed after 2 hours (black bars) or 96 hours (white bars), and colony-forming units (CFU) were enumerated at each time point. *B*, THP-1 macrophages were infected with *M. tuberculosis* strains H37Rv, SA161, M4100A, and SG1 for 48 hours and subsequently left untreated (UNT) or treated for an additional 48 hours with 40 μg/mL verapamil (+VER) before lysis and enumeration of CFU. Results are from 1 representative of ≥3 (*A*) or ≥2 (*B*) independent experiments, which are defined as experiments set up on different days with different cultures of bacteria and THP-1 cells. Error bars represent standard deviations. *P < .05, **P < .01, and ***P < .001, by the *t* test.

When we tested the 2 Beijing strains in our panel for their ability to grow in macrophages, we found that neither manifested an intramacrophage growth defect, confirming prior findings (Figure 2A). Thus, these Beijing strains have evolved compensatory mechanisms that allow them to grow in macrophages. Additional mechanisms that include host immune dysregulation have been invoked to further render them hypervirulent [7]. Indeed, when we assessed the SA161 Beijing strain in a mouse aerosol infection model, we found it to be hypervirulent. Transient, early increases in bacterial burdens as compared to H37Rv were associated with early lethality (Supplementary Figure 3). Together, these findings confirmed that SA161 not only has compensated for any macrophage growth defect due to the loss of Rv1258c, but also has further evolved additional mechanisms that render it hypervirulent. Because multiple efflux pumps are reported to be upregulated in Beijing strains [12], we considered the possibility that the mechanisms that compensated for its early growth in macrophages might include the induction of other efflux pumps. Consistent with this possibility, we found that the bacterial efflux pump inhibitor verapamil inhibited intramacrophage growth of SA161 to a degree similar to that observed for strains from other lineages [13] (Figure 2B).

## DISCUSSION

Our earlier studies showed that macrophage-induced drug tolerance is a potential contributor to the slow response of *M. tuberculosis* to antimicrobial treatment [2, 3]. However, the findings were limited to laboratory strains belonging to a single *M. tuberculosis* lineage. This work shows that macrophage-induced drug tolerance is a feature of the other 3 predominant *M. tuberculosis* lineages, as well. Moreover, the finding that the Beijing strains lack rifampin tolerance while retaining isoniazid tolerance corroborates our previous findings linking the Rv1258c efflux pump to the development of macrophage-induced rifampin tolerance [2].

The finding that the Beijing strains fail to develop macrophage-induced rifampin tolerance might be seen as presenting a potential quandary, given that Beijing lineage *M. tuberculosis* infection is more likely to relapse after treatment with standard rifampin-containing regimens [14]. However, this increased propensity to relapse may simply be due to the compensated growth in macrophages and hypervirulence traits of the Beijing lineage, as we have shown here for SA161. Furthermore, we demonstrate that treatment with the efflux pump inhibitor verapamil may inhibit intracellular growth of Beijing lineage strains even if they do not appear to develop macrophage-induced tolerance to rifampin.

Our finding that the majority of *M. tuberculosis* lineages responsible for disease worldwide exhibit macrophage-induced tolerance to rifampin suggests that strategies to inhibit efflux-mediated tolerance may be effective in shortening treatment regimens for the majority of patients. Although Beijing family strains do not demonstrate macrophage-induced tolerance to rifampin, efflux inhibition may still offer benefit for patients infected with these strains because verapamil, and possibly other efflux pump inhibitors, may reduce survival of Beijing family bacilli during the intracellular growth phase.

## Supplementary Data

Supplementary materials are available at *The Journal of Infectious Diseases* online. Consisting of data provided by the authors to benefit the reader, the posted materials are not copyedited and are the sole responsibility of the authors, so questions or comments should be addressed to the corresponding author.

Supplementary Figure 1

Supplementary Figure 2

Supplementary Figure 3

Supplementary Information Text

Supplementary Table 1

## Notes

***Acknowledgments.*** We thank Ian Orme for providing the SA161 strain, Shiva Kumar for providing the M1338 and NITR203 strains, Louie Galitan for technical assistance, and Paul Edelstein for providing input on the manuscript.

K. N. A., A. K. V., K. B. U., L. R., and R. E. H. designed experiments. K. N. A., A. K. V., R. G., H. A., D. K. S., and R. E. H. performed experiments. K. N. A., A. K. V., D. R. S., K. B. U., U. D. R., L. R., and R. E. H. analyzed and interpreted data. U. D. R., D. R. S., and S. T. provided project administration and supervision. K. N. A., L. R., and R. E. H. prepared figures and wrote the manuscript. All authors reviewed the manuscript. L. R. conceived the project.

***Financial support.*** This work was supported by the UK Medical Research Council (X5 06489 DBT-MRC Joint Centre Partnership) and the Department of Biotechnology, India (BT/IN/DBT-MRC (UK)/12/SS/2015-2016 for ICMR-National Institute for Research in Tuberculosis) as a Cambridge Chennai Partnership on Antimicrobial Resistant Tuberculosis, and by the National Institutes of Health (grants K08 AI116908 [to R. E. H.], T32 HD0073233 [to K. N. A.], and U19 AI135976 [to K. B. U. and D. R. S.]). LR is a Principal Research Fellow of the Wellcome Trust.

***Potential conflicts of interest.*** All authors: No reported conflicts of interest. All authors have submitted the ICMJE Form for Disclosure of Potential Conflicts of Interest. Conflicts that the editors consider relevant to the content of the manuscript have been disclosed.

Presented in part: Tuberculosis Drug Discovery and Development Gordon Research Conference, Lucca, Italy, June 2017; PacTB Symposium, Seattle, Washington, March 2018.
