# Supplementary figures and images for: Diverse Clinical Isolates of Mycobacterium tuberculosis Develop Macrophage-Induced Rifampin Tolerance
Source: J Infect Dis. 2019 Feb 7;219(10):1554–8. doi: 10.1093/infdis/jiy710 (PMC6473171; doi:10.1093/infdis/jiy710)

## Slide 1
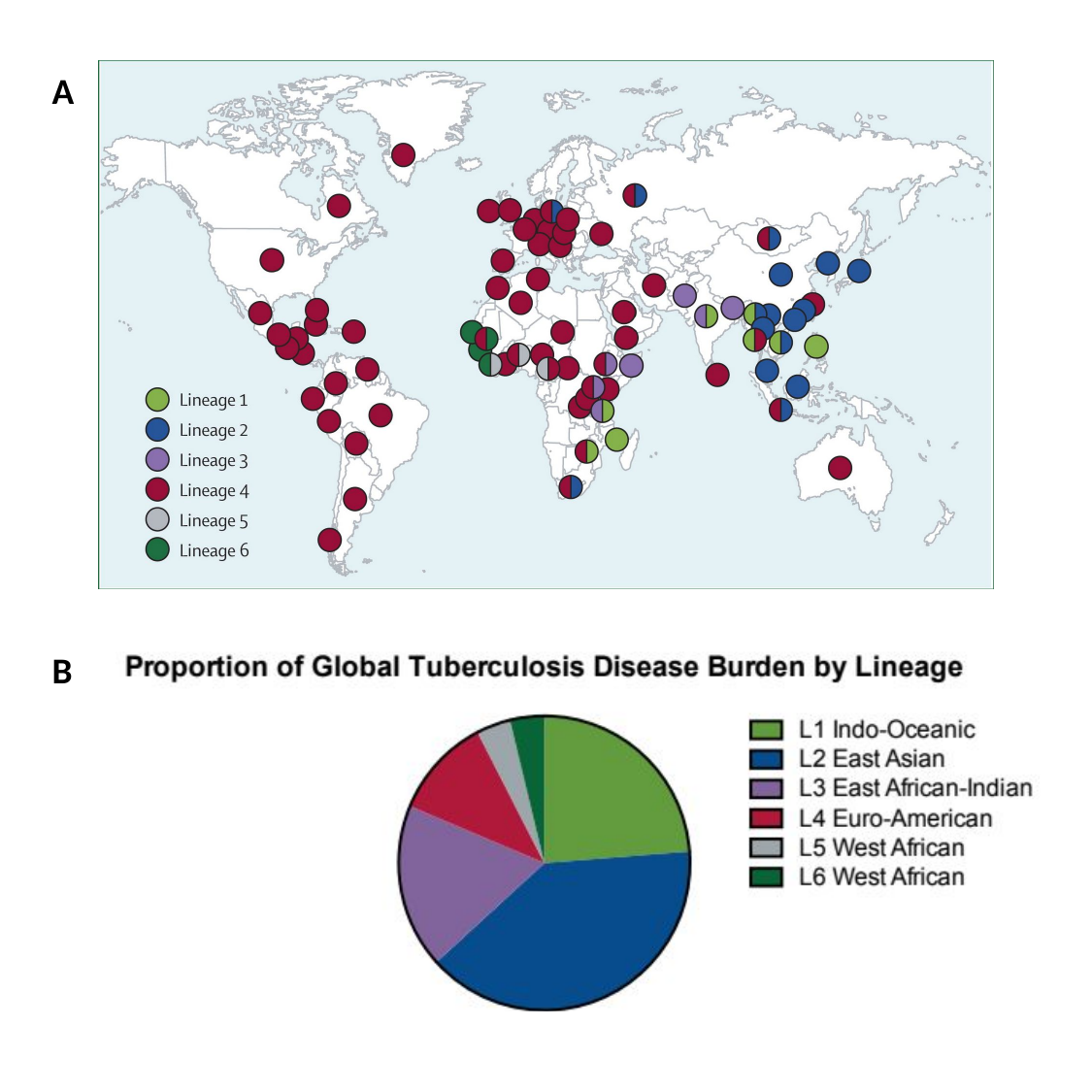

A
B

Supplement: Supplementary Figure 1 [file jiy710_suppl_supplementary-figure-1.pptx]

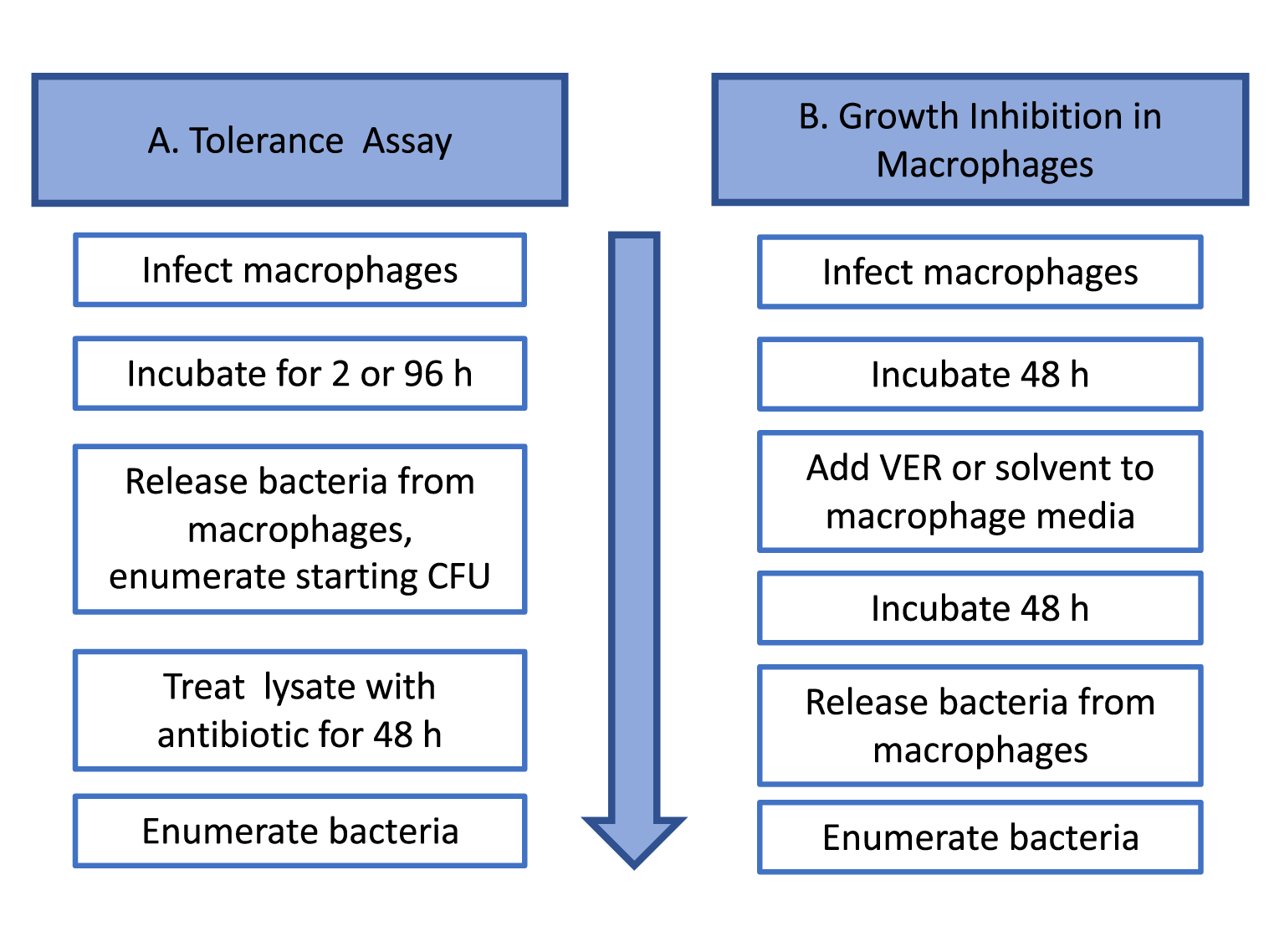

Supplement: Supplementary Figure 2 [file jiy710_suppl_supplementary-figure-2.png]

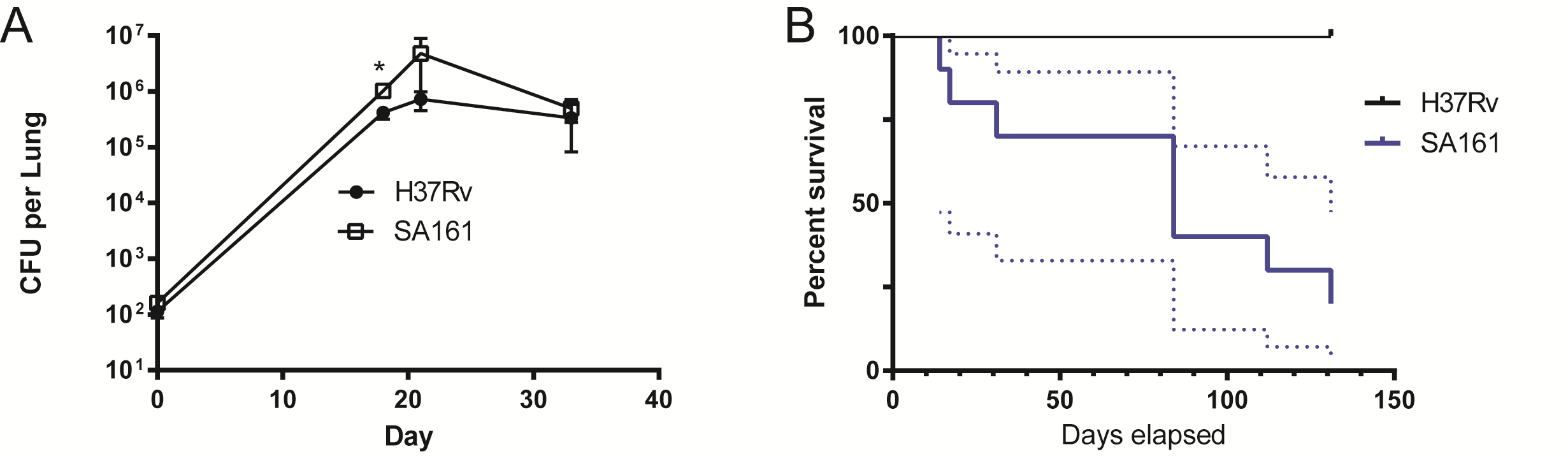

Supplement: Supplementary Figure 3 [file jiy710_suppl_supplementary-figure-3.png]
