# Supplementary material for: Diverse Clinical Isolates of Mycobacterium tuberculosis Develop Macrophage-Induced Rifampin Tolerance
Source: J Infect Dis. 2019 Feb 7;219(10):1554–8. doi: 10.1093/infdis/jiy710 (PMC6473171; doi:10.1093/infdis/jiy710)
Supplement: Supplementary Information Text [file jiy710_suppl_supplementary_information_text.docx]

**Supplementary Information**

**Supplementary Materials and Methods**

*Mice and aerosol infections*

All animals were housed and maintained in specific pathogen free conditions at Center for Infectious Disease Research (CIDR). Age and sex matched C57BL/6 mice were purchased from Jackson Laboratories (Bar Harbor, ME). All animal studies were conducted in accordance with an animal study protocol approved by CIDR Animal Care and Use Committee. Male and female mice between the ages of 8-12 weeks were used for experiments.

Bacterial strains used for animal infections were: H37Rv and SA161. Mice were infected with ~50-100 CFU of aerosolized *Mycobacterium tuberculosis* (Mtb) in a Glas-Col infection chamber (Glas-Col, Terre Haute, IN). Two mice from each infection were sacrificed and lung homogenates were plated on 7H10 agar to determine initial deposition of colony forming units (CFU). Bacterial burdens were determined from five mice at all later time points by plating whole lung homogenate on 7H10 agar and counting CFU after three weeks growth. For the survival analysis, mice were infected with approximately 250 CFU. As an alternate humane end point, animals showing weight loss of 20% were euthanized.

*Minimum Inhibitory Concentration (MIC) assays*

MICs were determined my microbroth dilution, as described [1]. Briefly, approximately 10^4^ bacteria in 100 μl were added to round bottom 96 well plates containing 100 μl of drug-supplemented 7H9 lacking Tween-80. The plates were incubated at 37^o^C for 6-8 days, prior to the addition of 32.5 μl sodium resazurin (0.02% in water with 7.7% Tween80) for one day. The lowest concentration of antibiotic that inhibited a visible change color was defined as the MIC.

*Confirmation of Tap^580^insert in Rv1258c*

Lineage 2 Beijing strains are reported to carry a frameshift mutation in *Rv1258c*. *Rv1258c* from clinical strains was amplified by PCR using primers as previously described [2], followed by digestion with XhoI. The insertion was also confirmed to be present in NIRT203 and SA161 by whole genome sequencing.

**Supplementary Figure Legends**:

**Supplementary Figure 1: Global tuberculosis distribution and disease burden by *Mycobacterium tuberculosis* complex lineage.** A. The global distribution of dominant MTB lineages in each country estimated in 2002 (reprinted from [7], with permission from Elsevier; figure previously adapted from [8], Copyright 2006 National Academy of Sciences). B. The proportion of total global cases of tuberculosis attributed to each of the major 6 MTB lineages, figure drawn from data presented in [7].

**Supplementary Figure 2:** **Schematic of experimental procedures**. A. The ability of MTB strains to develop macrophage induced tolerance was assessed by infecting THP1 macrophages at a multiplicity of infection of 1. At 2 h or 96 h post-infection macrophages were lysed and the colony forming units at the time of lysis (CFU) was determined by plating. Aliquots of bacteria in the macrophage lysate were then treated with antibiotics for 48 hours, before plating dilutions to determine the fraction of bacteria surviving antibiotic treatment. B. Inhibition of intracellular growth by verapamil was determined by infecting THP1 macrophages for 48 h, then adding verapamil HCl (VER, 40 μg/mL) or solvent (water) to the RMPI based media, incubating for an additional 48 h, then lysing the THP1 macrophages to determine CFU by plating.

**Supplementary Figure 3: The lineage 2 Beijing strain, SA161, demonstrates similar growth in the mouse lung to H37Rv but is markedly hypervirulent.** A. C57BL/6 mice were infected with approximately 100 CFU of either H37Rv or SA161 and colony forming units (CFU) in the lung were measured at days 18, 21 and 33 days post-infection. Representative from three experiments. Mean CFU at each time point were compared by t-test, with Holm-Sidak correction for multiple comparisons. Error bars represent standard deviation. B. 8-9 C57BL/6 mice were infected with approximately 250 CFU of either H37Rv or SA161 and monitored for 135 days post-infection. Dashed lines represent 95%-confidence intervals. Results from one experiment. Significance determined by Log-rank (Mantel-Cox) test.

**Supplementary References:**

1. Palomino J-C, Martin A, Camacho M, Guerra H, Swings J, Portaels F. Resazurin microtiter assay plate: simple and inexpensive method for detection of drug resistance in Mycobacterium tuberculosis. Antimicrobial Agents and Chemotherapy. **2002**; 46:2720–2722.

2. Villellas C, Aristimuno L, Vitoria MA, et al. Analysis of Mutations in Streptomycin-Resistant Strains Reveals a Simple and Reliable Genetic Marker for Identification of the Mycobacterium tuberculosis Beijing Genotype. Journal of Clinical Microbiology. **2013**; 51:2124–2130.

3. Shanmugam S, Selvakumar N, Narayanan S. Drug resistance among different genotypes of Mycobacterium tuberculosis isolated from patients from Tiruvallur, South India. Infect Genet Evol. **2011**; 11:980–986.

4. Narayanan S, Deshpande U. Whole-Genome Sequences of Four Clinical Isolates of Mycobacterium tuberculosis from Tamil Nadu, South India. Genome Announc. **2013**; 1:e00186–13–e00186–13.

5. Comas I, Coscolla M, Luo T, et al. Out-of-Africa migration and Neolithic coexpansion of Mycobacterium tuberculosis with modern humans. Nat Genet. **2013**; 45:1176–1182.

6. Stucki D, Malla B, Hostettler S, et al. Two new rapid SNP-typing methods for classifying Mycobacterium tuberculosis complex into the main phylogenetic lineages. PloS one. **2012**; 7:e41253.

7. Gagneux S, Small PM. Global phylogeography of Mycobacterium tuberculosis and implications for tuberculosis product development. Lancet Infect Dis. **2007**; 7:328–337.

8. Gagneux S, DeRiemer K, Van T, et al. Variable host-pathogen compatibility in Mycobacterium tuberculosis. Proceedings of the National Academy of Sciences of the United States of America. **2006**; 103:2869–2873.
