# Supplementary material for: Diverse Clinical Isolates of Mycobacterium tuberculosis Develop Macrophage-Induced Rifampin Tolerance
Source: J Infect Dis. 2019 Feb 7;219(10):1554–8. doi: 10.1093/infdis/jiy710 (PMC6473171; doi:10.1093/infdis/jiy710)
Supplement: Supplementary Table 1 [file jiy710_suppl_supplementary_table-1.docx]

**Supplementary Table 1**

| **Strain Name** | **Genetic Lineage** | **Obtained from** | **MIC (μg/ml)** | | **Presence/absence of Tap580 insertion & method of confirmation** | **Reference** |
| --- | --- | --- | --- | --- | --- | --- |
|  |  |  | RIF | INH |  |  |
| H37Rv | 4 | C Sassetti  NIRT | 0.15  0.4* | 0.08  0.2* | PCR/XhoI digest |  |
| CDC1551 | 4 | WR Bishai | 0.075 | 0.08 | Genome sequencing and PCR/XhoI digest |  |
| M1338 | 1 | NIRT Clinical Isolate | 0.4* | 0.2* |  | [3] |
| NITR203 | 2 Beijing | NIRT Clinical Isolate | 0.4* | >1.6* | Genome sequencing | [4] |
| SA161 | 2 Beijing | I Orme | 0.15 | 0.16 | Genome sequencing and PCR/XhoI digest |  |
| M4100A | 2 non-Beijing | S Gagneux | 0.075 | 0.04 | PCR/XhoI digest | [5,6] |
| SG1 | 3 | S Gagneux | 0.075 | 0.08 | PCR/XhoI digest | [5] |
| GM1503 | 4 | S Gagneux | 0.08 | 0.03 | PCR/XhoI digest | [5] |

*MIC determined at the National Institute for Research in Tuberculosis in Chennai, India
